# Multimodal Preoperative Management of Rectal Cancer: A Review of the Existing Guidelines

**DOI:** 10.3390/medicina61071132

**Published:** 2025-06-24

**Authors:** Ionut Negoi

**Affiliations:** 1 Department of General Surgery, Faculty of Medicine, Carol Davila University of Medicine and Pharmacy Bucharest, 050474 Bucharest, Romania; ionut.negoi@umfcd.ro; 2 Clinical Emergency Hospital of Bucharest, 014461 Bucharest, Romania

**Keywords:** rectal cancer, multimodal preoperative management, total neoadjuvant therapy, immunotherapy, mismatch repair status

## Abstract

Rectal cancer management necessitates a rigorous multidisciplinary strategy, emphasizing precise staging and detailed risk stratification to inform optimal therapeutic decision-making. Obtaining an accurate histological diagnosis before initiating treatment is essential. Comprehensive staging integrates clinical evaluation, thorough medical history analysis, assessment of carcinoembryonic antigen (CEA) levels, and computed tomography (CT) imaging of the abdomen and thorax. High-resolution pelvic magnetic resonance imaging (MRI), utilizing dedicated rectal protocols, is critical for identifying recurrence risks and delineating precise anatomical relationships. Endoscopic ultrasound further refines staging accuracy by determining the tumor infiltration depth in early-stage cancers, while preoperative colonoscopy effectively identifies synchronous colorectal lesions. In early-stage rectal cancers (T1–T2, N0, and M0), radical surgical resection remains the standard of care, although transanal local excision can be selectively indicated for certain T1N0 tumors. In contrast, locally advanced rectal cancers (T3, T4, and N+) characterized by microsatellite stability or proficient mismatch repair are optimally managed with total neoadjuvant therapy (TNT), which combines chemoradiotherapy with oxaliplatin-based systemic chemotherapy. Additionally, tumors exhibiting high microsatellite instability or mismatch repair deficiency respond favorably to immune checkpoint inhibitors (ICIs). The evaluation of tumor response following neoadjuvant therapy, utilizing MRI and endoscopic assessments, facilitates individualized treatment planning, including non-operative approaches for patients with confirmed complete clinical responses who comply with rigorous follow-up. Recent advancements in molecular characterization, targeted therapies, and immunotherapy highlight a significant evolution towards personalized medicine. The effective integration of these innovations requires enhanced interdisciplinary collaboration to improve patient prognosis and quality of life.

## 1. Introduction

Rectal cancer is a common cancer worldwide that significantly impacts morbidity, mortality, and healthcare resources. During the last three decades, globally, the age-standardized incidence rate of colorectal cancer has increased from 22.2 to 26.7 per 100,000, and the Disability-Adjusted Life Years (DALYs) attributed to this disease have increased from 12.4 million to 24.3 million [1]. Advancements in preoperative diagnostic imaging, chemoradiotherapy, and surgical methods have improved survival rates and enhanced quality of life. However, managing rectal cancer is complex and requires a multidisciplinary approach that incorporates accurate staging, risk assessment, and individualized treatment plans [2].

For patients with locally advanced rectal cancer, the standard treatment protocol involves a combination of chemotherapy and radiotherapy before surgery. Recent studies have reinforced the adoption of total neoadjuvant therapy (TNT), which has shown improved tumor response rates and the possibility of organ preservation [3]. Furthermore, the development of targeted therapies and immunotherapy guided by molecular profiling, such as microsatellite instability (MSI) and mismatch repair (MMR) status, has paved the way for more personalized treatment strategies.

This study aimed to review the current recommendations regarding diagnostic imaging, neoadjuvant therapies, response evaluation, and personalized treatments based on molecular profiling to promote consistency in clinical practice.

## 2. Methods

This umbrella review systematically evaluated existing guidelines, systematic reviews, meta-analyses, and consensus documents addressing multimodal preoperative management of rectal cancer [4,5]. A literature search was conducted using the Web of Science and PubMed/MEDLINE databases. The search strategy used in the Web of Science Core Collection was: ((TI = (rectal cancer)) OR TI = (rectal adenocarcinoma) OR TI = (rectal neoplasm)) AND ((TI = (guideline) OR TI = (guidance))).

The inclusion criteria were as follows: (1) guidelines, systematic reviews, meta-analyses, or consensus statements; (2) focus on preoperative rectal cancer management; (3) explicit recommendations for diagnosis and treatment; (4) full-text availability; (5) English language. Case reports, editorials, studies exclusively on colon cancer, animal studies, and low-quality articles were excluded.

The retrieved outcomes were those with prognostic and predictive value, the accuracy of imaging methods in different clinical scenarios, and clinical oncological outcomes.

A critical appraisal of the methodological quality of the included guidelines was not performed, considering that they were produced by the most prestigious societies of colorectal surgery worldwide, and the articles were published in high-impact journals.

Data extraction was standardized to capture details of the diagnostics, therapeutic strategies, patient selection, response evaluation, and multidisciplinary recommendations. Recommendations were qualitatively synthesized by comparing and categorizing the findings, highlighting the consensus and conflicts, and examining methodological differences and clinical contexts. Ethical approval was not required for this study, as it involved only the published literature without any patient data. Microsoft Office version 2024 for Mac, Web of Science, Perplexity, and ChatGPT were used as artificial intelligence software programs for the conceptualization, investigation, review, and editing of the manuscript [6].

## 3. Results

**Figure 1:**
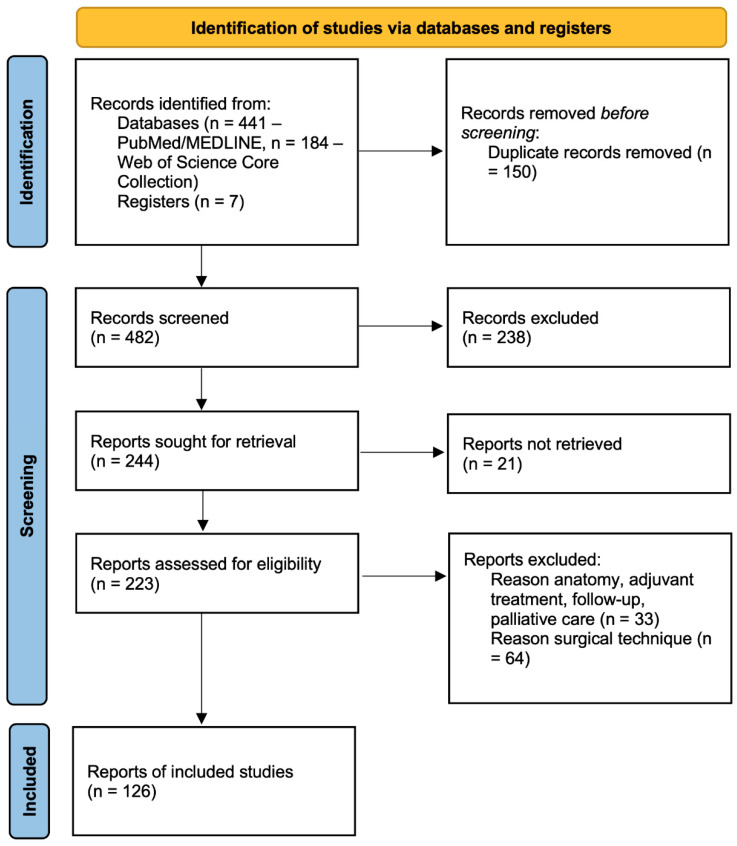
PRISMA flow diagram.

We screened 482 titles and abstracts (Figure 1). We included 126 studies in the present qualitative synthesis.

### 3.1. Clinical Examination

A histological diagnosis of rectal cancer prior to therapeutic intervention is essential. Exceptions should be rare in cases of indeterminate biopsies, lesions that cannot be resected because of their large dimensions [7], or acute complications that are not responsive to conservative treatment. The patient’s history should be carefully screened for increased familial risk, the clinical examination should look for signs and symptoms of the disease and evaluate overall surgical risks, CEA should be monitored, and CT of the abdomen and thorax should be mandatory for staging [8]. Clinical examinations should determine the proximity of the tumor to the anal verge and evaluate its relationship with the sphincter complex.

### 3.2. Imaging

For patients diagnosed with rectal cancer, a high-resolution pelvic MRI using a specialized rectal protocol is recommended before initiating treatment (Table 1). This imaging technique serves two crucial purposes: assessing the risk factors for cancer recurrence and providing essential information for developing an appropriate treatment strategy. A standardized MRI report is recommended that provides details on how the primary tumor relates to various anatomical structures and features, such as its position relative to the anal verge, the sphincter complex, and the pelvic nodes. The report should also describe the relationship between the tumor and the mesorectal fascia, the presence of extramural vascular invasion (EMVI), tumor deposits, and lymph nodes [9,10].

MRI demonstrated high precision in detecting circumferential resection margin (CRM) involvement, with a specificity of 94% (95% CI, 88–97%). This surpassed the specificity for T-category assessment (75%, 95% CI 68–80%) and lymph node evaluation (71%, 95% CI 59–81%). Consequently, MRI is an effective technique for assessing future surgical resection margins [16].

In an Australian study examining the effectiveness of MRI for staging rectal cancer without preoperative chemoradiation, the overall concordance between MRI findings and final pathology results was 55% for the T stage and 65% for the N stage. The MRI accuracies were 91%, 43%, 65%, and 80% for T1, T2, T3, and T4 tumors, respectively. For the N stage, the accuracies were 74.1% and 44.4% for N-negative and N-positive tumors, respectively [17].

A meta-analysis of 37 studies found that preoperative MRI had a sensitivity of 0.73 (95% CI 0.68–0.77), specificity of 0.74 (95% CI 0.68–0.80), and diagnostic odds ratio of 7.85 (95% CI 5.78–10.66) [18]. The criteria for positive lymph nodes were as follows: (a) short diameter > 5 mm; (b) abnormal morphology, including irregular shape and mixed central signal; (c) a + b; (d) short axis > 8 mm + abnormal morphology; (e) short diameter > 10 mm and abnormal morphology. The sensitivities were 75%, 81%, 74%, 72%, and 62%, and the specificities were 64%, 67%, 79%, 66%, and 91%, respectively [18].

An analysis of 5539 patients from the Dutch ColoRectal Audit with cT1–T2 tumors treated with local excision or TME showed substantial overstaging on preoperative MRI [19]. For patients diagnosed with pT1 disease, MRI alone resulted in overstaging in 54.7% of cases, whereas the combination of MRI and EUS led to overstaging in 31% of cases. In contrast, understaging of pT2 occurred in 8.2% of patients when using MRI alone and in 27.9% when using both MRI and EUS. Regarding patients with pT1N0 disease, MRI accurately staged 30.3% of cases while incorrectly classifying 58% as cT2N0 and 11.6% as cT1,2N1 [19].

A study examining 1888 patients from the Swedish Colorectal Cancer Registry who underwent surgical resection for rectal cancer between 2009 and 2018 and had MRI T1–2 tumors revealed that the accuracy of MRI for cT1–T2 tumors was 67.9%. This study uncovered a significant understaging of T3 tumors, which accounted for 30% of the cT1–T2 tumors. Furthermore, the majority (74.2%) of pN+ tumors were incorrectly classified as cN0, whereas most cN+ tumors (56.2%) were actually pN0 [20].

For restaging after neoadjuvant chemoradiotherapy, MRI had positive predictive values (PPVs) of 50–67% and 54–62% for T1–2 and T3–4, respectively, and negative predictive values (NPVs) of 72–90% and 33–78%, respectively [21]. For N0, the PPV was 81–95%, and the NPV was 58–73% [21].

In early-stage rectal cancer, endoscopic ultrasound (EUS) can be used to determine the extent of tumor invasion and distinguish between the T1 and T2 stages.

The accuracy of endorectal ultrasound ranges from 62% to 92% for T stage assessment and 64% to 88% for N stage evaluation, according to previous studies [22,23,24].

An analysis of 42 studies demonstrated that endoscopic ultrasound (EUS) exhibited sensitivities of 87.8%, 80.5%, 96.4%, and 95.4% for identifying the T1, T2, T3, and T4 stages, respectively. The corresponding specificities were 98.3%, 95.6%, 90.6%, and 98.3%, respectively [25].

The accuracy of EUS is influenced by tumor location and stage. Lower rectal tumors are more likely to be incorrectly staged than those from the middle and upper rectum (16.7% versus 6,3%, *p* < 0.001) [22]. Furthermore, EUS tends to overestimate the stage of early tumors (T1) and underestimate the stage of more advanced tumors (T3) [26].

The accuracy of EUS decreases after neoadjuvant chemoradiotherapy, with a tendency to overstage tumors owing to treatment-induced changes in tissue characteristics [27,28].

Preoperative full colonoscopy should be performed to investigate synchronous colonic tumors; in the case of intestinal obstruction, abdominal CT and intraoperative revaluation should be completed with colonoscopy within the following 6 months [8].

Synchronous colorectal cancer occurs at a frequency of 1–8% [29]. A systematic review of computed tomography colonography (CTC) for identifying synchronous colorectal cancer (CRC) revealed a detection rate of 5.7%. This meta-analysis included 21 studies published between 1997 and 2018. In contrast, a separate meta-analysis focusing on non-CTC diagnostic methods, which encompassed 27 studies conducted between 1974 and 2015 and involved 111,873 patients, reported a synchronous CRC detection rate of 3.9% [30].

An analysis of 10 years from the Dutch ColoRectal Audit showed a 3.1% rate of synchronous rectal and colonic resections among 100,474 patients [31]. Synchronous bilateral colorectal resection, which involved subtotal colectomy in 25.4% of cases, was correlated with increased rates of postoperative complications, subsequent surgical interventions, prolonged hospitalization, and increased mortality [31].

### 3.3. Multidisciplinary Team (MDT)

A multidisciplinary team (MDT) discussion is required for each rectal cancer case to determine the optimal treatment strategy. Qualified surgeons with sufficient case loading, experience, expertise, and proper training should perform the surgical procedure.

Rectal cancer treatment has undergone substantial advancements, with a collaborative interdisciplinary team approach playing a crucial role in achieving the best possible outcomes [32]. This approach involves collaboration among various specialists, including radiologists, oncologists, surgeons, and pathologists, to ensure comprehensive care. MDTs provide a platform for robust research and innovation governance. These are essential for integrating new treatment modalities and tailoring therapies based on clinicopathological features and the identification of emerging molecular and genetic markers [33,34]. The MDT approach is associated with improved survival and reduced morbidity rates. This allows the implementation of advanced surgical techniques and multimodal therapies, which are critical for managing stage II and III cancers [35,36,37]. By following standardized protocols and evidence-based pathways, MDTs can ensure high-quality care and reduce treatment outcome disparities. This is particularly important in regions with high variability in cancer care, such as European countries [36,38,39].

MDTs improve coordination and communication among healthcare professionals, which is crucial given the complexity of rectal cancer treatment. Studies have demonstrated that this approach decreases variations in care and enhances patient outcomes by promoting collaborative, evidence-driven decision-making in treatment planning [36,37,40,41]. A global assessment showed that the MDT substantially impacted decisions regarding MRI (risk ratio [RR] = 3.62), neoadjuvant therapy for threatened CRM (RR = 5.67) or advanced local stages (RR = 2.98), pathology report quality (RR = 4.85), and sphincter-preserving surgical procedures (RR = 3.81) [41].

MDT plays a crucial role in improving the decision-making process and optimizing treatment strategies, particularly for complex conditions such as locally advanced and recurrent rectal cancers. These teams can tailor treatment plans to individual patient needs by incorporating the latest advances in imaging, surgery, and chemoradiotherapy [33,42,43]. An examination of 696 colorectal cancer (CRC) cases in Italy, evaluated by a multidisciplinary team (MDT), revealed that 214 decisions were changed following MDT discussions. This resulted in an overall discrepancy rate of 31% [44]. Imaging evaluations were modified in 29% of cases, and there was a 73% discrepancy for 80 cases initially defined as progressed and stable after MDT. The surgical treatment strategy was changed after MDT in 35% of cases, with 19% of cases defined before MDT as unresectable and reconsidered as resectable after MDT [44].

Although the benefits of MDTs are clear, challenges remain in ensuring consistent implementation and compliance with their recommendations. Factors such as hospital volume and patient comorbidities can influence the likelihood of MDT evaluation and adherence to treatment plans [38,43].

### 3.4. Microsatellite Instability and Mismatch Repair Deficiency

Microsatellite instability (MSI) testing in rectal cancer has significant clinical implications, particularly in predicting treatment response and prognosis. MSI is a genetic feature that can influence the rectal cancer response to therapies, especially neoadjuvant chemoradiation [45]. Patients with rectal cancer may undergo an assessment of MSI or mismatch repair (MMR) status before initiating therapy [9].

Microsatellite instability indicates that the number of repeated DNA bases within a short DNA sequence, known as a microsatellite, differs from the inherited number [46]. MSI is a direct consequence and key marker of DNA mismatch repair deficiency (dMMR) [47]. The core MMR machinery, which plays a critical role in rectifying errors that occur during DNA replication within a cell, consists of proteins encoded by the MLH1, MSH2, MSH6, and PMS2 genes [46,48,49]. Mutations in these genes may be classified as either germline, as observed in Lynch syndrome (accounting for approximately 3% of all colorectal cancers), or sporadic, which constitutes approximately 12% of colorectal cancer cases [50]. In clinical practice, the MSI or dMMR status can be evaluated using two complementary methods: immunohistochemistry and genetic analysis. Immunohistochemical analysis identified the presence of proteins encoded by the PMS2, MLH1, MLH2, and MSH6 genes. The absence of these proteins suggests a mutation in the corresponding genes. Genetic analysis involves the use of polymerase chain reaction (PCR) or next-generation sequencing (NGS) for the direct identification of these four genes. A tumor is classified as having high microsatellite instability (MSI-H) when mutations are detected in > 30% of microsatellites [51].

MSI-H rectal cancer is recognized as a relatively rare molecular subtype within the broader category of rectal cancers. Studies have indicated that MSI-H tumors account for approximately 1.5–6.3% of all rectal cancer cases. Emile et al. reported that 6.3% of their studied rectal cancer population was MSI-H [51], while another study by Lee et al. found a lower incidence of 1.5% [52]. Furthermore, other literature reviews suggest that the representation of MSI-H in rectal cancers is lower than that in colon cancers, although the proportion varies across studies [51,52].

However, it should be noted that neoadjuvant chemoradiotherapy may change the MSI status of rectal tumors and that pre-chemoradiotherapy, but not post-chemoradiotherapy, MSI status correlates with the response to neoadjuvant chemoradiotherapy [53]. Bae et al. observed changes in MSI status in 11 out of 60 patients, from microsatellite stable (MSS) to MSI-low in nine cases, from MSS to MSI-high in one patient, and from MSI-low to MSS in one patient [53]. The Mandard tumor regression grades (TRGs) were 1 or 2 (responders) in 25 of 84 patients (29.8%) and 3, 4, and 5 (non-responders) in 59 patients (70.2%). A significant association was found between TRG and MSI status before CRT (*p* = 0.024); however, no such correlation was observed with MSI status after CRT (*p* = 0.788) [53].

MSI-positive rectal cancers exhibit a reduced frequency of pathological complete response (pCR) to preoperative chemoradiation compared with MSI-negative cancers. Studies have shown that MSI-positive patients have a pCR rate of 5.9%, compared with 8.9% in MSI-negative patients, indicating a reduced response to treatment [54,55]. A study of the National Cancer Database, which included 5086 patients (636 MSI-positive and 4450 MSI-negative) diagnosed with locally advanced rectal cancer between 2010 and 2015 who underwent chemoradiation and surgery, revealed that T stage and MSI positivity were notably linked to a decreased pCR rate (OR = 0.65, 95% CI 0.43 to 0.96) [54]. Patients who achieved pCR had a 93% 5 year survival rate compared with 73% for those who did not achieve pCR (*p*< 0.001) [54].

Although MSI status serves as a powerful prognostic indicator in colorectal cancer, its influence on rectal cancer survival is unclear. Research has indicated that an MSI-high status may be correlated with improved overall survival in patients with colorectal cancer. However, this relationship has not been consistently observed in rectal cancer [56,57].

Swets et al. examined the effect of MSI status on patients with rectal cancer. Their study incorporated data from two prospective phase III randomized trials (TME and PROCTOR-SCRIPT trials, encompassing 1250 participants) and findings from an additional 21 studies identified through a comprehensive literature review [58]. Of the 16,526 patients examined, 1220 exhibited high microsatellite instability (MSI), representing 6.7% of the sample, with a standard error of 1.19%. The study found that MSI status did not significantly influence overall survival (hazard ratio [HR] = 1, 95% confidence interval [CI] 0.77–1.29) or disease-free survival (HR = 0.86, 95% CI = 0.60–1.22). Additionally, MSI status showed no substantial effect on downstaging (risk ratio [RR] = 1.15, 95% CI: 0.86–1.55) or pathological complete response (RR = 0.81, 95% CI: 0.54–1.22) [58].

MSI tumors are often infiltrated by lymphocytes, which can influence their response to immune checkpoint inhibitors (ICIs). The potential of MSI as a predictive marker for immunotherapy response is implied (see below), although further research is necessary to elucidate its specific role in rectal cancer [53].

MSI testing can help stratify patients for personalized treatment. For example, individuals with MSI-positive neoplasms may respond better to alternative treatment approaches than those with MSI-negative neoplasms [59,60].

Immunotherapy is an emerging treatment modality for rectal cancer, especially in individuals with specific genetic characteristics, such as high microsatellite instability (MSI-H) or deficiency in mismatch repair (dMMR) tumors.

### 3.5. Testing in M1 Disease

In patients with rectal cancer and potential metastases, genetic testing can be performed using tissue samples or blood-based next-generation sequencing (NGS). This analysis can detect mutations in RAS (including KRAS and NRAS) and BRAF genes, as well as HER2 amplification and alterations in POLE/POLD1, RET, and NTRK genes [61,62].

Mutations in the RAS genes significantly influence the management of metastatic rectal cancer, particularly in determining the potential success of anti-EGFR therapy. Mutations in RAS genes, specifically KRAS and NRAS, occurring in exons 2 (codons 12 and 13), 3 (codons 59 and 61), and 4 (codons 117 and 146), are associated with the ineffectiveness of anti-EGFR monoclonal antibodies. Patients with these mutations do not respond favorably to treatments such as cetuximab, whether administered alone or in combination [63,64,65,66].

A meta-analysis conducted by Peeters et al. examined five randomized studies, including 3196 cases of metastatic colorectal cancer from 36 countries. Their findings indicated an overall prevalence of RAS mutations of 55.9%. KRAS mutations were observed in exons 2 (42.6%), 3 (3.8%), and 4 (6.2%). Additionally, NRAS mutations were found in exons 2 (2.9%), 3 (4.2%), and 4 (0.3%) [67].

Although RAS mutations are highly prevalent in colorectal cancer (approximately 40% KRAS and 4% NRAS), until recently, they were considered ”undruggable” [68]. However, novel KRAS^G12C^ mutation inhibitors have shown promising results, and their combination with anti-EGFR drugs or immunotherapy offers new therapeutic possibilities [68].

CRCs with BRAF mutations present significant therapeutic challenges and are associated with poor prognosis. BRAF V600E mutation is observed in 8–15% of CRCs, while non-V600 BRAF mutations account for 2% of cases [69,70,71,72].

Jiang et al. showed that BRAF mutations are associated with resistance to neoadjuvant chemoradiotherapy, with shorter progression-free and overall survival in patients with locally advanced disease [73].

Recently, BRAF V600E has become an actionable target in colorectal cancer [74]. Recent evidence has demonstrated the advantages of using BRAF-targeted therapies, either alone or in combination with MEK inhibition and epidermal growth factor receptor-targeted treatment, in patients diagnosed with advanced colorectal cancer carrying the BRAF V600E mutation [74]. Additionally, it is important to identify patients with MSI-H/dMMR and BRAF V600E mutations, as this subgroup may respond favorably to immune checkpoint inhibitors [72,74,75].

HER2 overexpression or amplification is relatively rare in rectal cancer, with studies reporting prevalence rates ranging from 2.2% to 5.8% [76,77]. HER2-targeted therapies, such as dual HER2 blockade (monoclonal antibodies such as trastuzumab and pertuzumab) or combinations of monoclonal antibodies with tyrosine kinase inhibitors (trastuzumab and lapatinib), have shown promise in a subset of metastatic colorectal cancer (CRC) cases, particularly in those resistant to standard therapies [78].

PET/CT is indicated in patients with potentially resectable metastatic disease [61].

PET/CT may be recommended in specific scenarios, such as suspected metastatic disease at presentation, recurrence workup, and contraindications to contrast agents [79].

PET/CT is an efficient method for identifying extrahepatic disease (EHD) in patients with colorectal liver metastasis (CRLM) and can affect treatment decision-making. Studies have demonstrated that this method identifies more EHD cases than traditional imaging techniques, such as CT and MRI. This detection ability can result in modifications to treatment approaches in approximately 25% of patients [80,81]. Although PET/CT provides comparable sensitivity to CT for detecting intrahepatic metastases, it is superior in identifying recurrent intrahepatic tumors after hepatectomy and local recurrences at the primary colorectal site [81]. PET/CT may change the surgical approach in a subgroup of patients. In randomized trials, PET/CT altered surgical plans in approximately 8% of patients, whereas non-randomized studies reported changes in 20% of patients [82,83]. The PETCAM trial indicated that PET/CT did not significantly affect the 5 year DFS or OS, although it resulted in a higher proportion of major liver resections [83,84,85]. PET/CT plays an important role in identifying metastatic rectal cancer and is particularly effective in detecting metastatic lymph nodes. It offers high specificity and accuracy, making it a useful tool for clinical decision-making. PET/CT exhibited 86 overall accuracy of 86% in the detection of metastatic lymph nodes, with a sensitivity of 80% and specificity of 91% [86]. Another study reported an accuracy of 90.1% for PET/CT in diagnosing metastatic lymph nodes using a specific diagnostic threshold [87]. For detecting pelvic recurrence after resection, PET/CT has a high sensitivity and specificity of 98% and 96%, respectively [88].

### 3.6. Early Rectal Cancer Treatment

In cases of early rectal cancer (T1–T2, N0, and M0), the standard treatment approach is radical surgery [89,90,91]. Preoperative radiotherapy should not be offered to these patients unless they are part of a clinical trial [62]. T1N0 tumors may be resected through transanal local excision (transanal minimally invasive surgery and transanal endoscopic microsurgery, as non-laparoscopic, non-endoscopic techniques) if the tumor has the following characteristics: (i) appropriate/suitable location within the lower 8 cm of the rectum measured from the anal verge, mobile, size not exceeding 3 cm, involving less than 30% of the circumference, full-thickness excision seems reasonable, and resection margins > 3 mm; (ii) good pathological features: no lymph node involvement, no poor differentiation, no lymphovascular invasion, or perineural invasion. T2N0 tumors: radical surgery is indicated [91].

Transanal full-thickness local excision could be an option for T1N0 cancers or those with a near-complete response and a strong but incomplete response that are not able to undergo or refuse radical surgery [61].

The tumor location is indicated to be distal to the second Houston valve, which usually corresponds to the intraperitoneal rectum [14].

This procedure is not indicated for patients with cCR. TEM, TEO, and TAMIS are preferred over traditional per-anal excision, especially for more proximal lesions. Tumor fragmentation should be avoided, and negative margins (> 3 mm) are required. If the local resection specimen presents adverse features, such as invasion of the deepest third of the submucosal layer (sm3), positive margins, poor differentiation, or lymphovascular invasion, a more complex resection is required [10].

Endoscopic submucosal resection (ESD) may be considered for T1 lesions that are well or moderately differentiated, with no lymphovascular invasion and a submucosal invasion depth of less than 1000 µm, and should be performed in high-volume centers [61].

### 3.7. Locally Advanced Cancers

In locally advanced rectal cancers (T3, T4, and N+) with microsatellite stability or proficient mismatch repair mechanisms, the initial therapeutic approach should include total neoadjuvant therapy (TNT). This treatment strategy, which combines chemoradiotherapy (CRT) and oxaliplatin-based chemotherapy, is usually indicated for tumors located in the lower rectum, as determined by MRI, and/or for those at an increased risk of developing local and/or distant recurrence. Factors indicating a higher risk include T4 stage, extramural vascular invasion (EMVI), tumor deposits, affected mesorectal fascia, and a compromised intersphincteric plane [9]. TNT is particularly recommended for patients aiming to increase their chances of cCR [92]. On the other hand, studies indicate that patients undergoing TNT experience higher incidences of postoperative complications, including bowel, urinary, and sexual dysfunction, which can significantly impact their quality of life. For example, García-Aguilar et al. found that the multimodal nature of TNT contributes to considerable treatment-related morbidity, including impairments in bowel, urinary, and sexual functions [93,94].

In patients who qualify for TNT, chemotherapy administration after radiation is the recommended approach. Although neoadjuvant long-course CRT is typically preferred over short-course RT, the latter may be considered an appropriate treatment alternative based on the specific circumstances of the patient.

In cases of locally advanced rectal cancer (T3, T4, and/or N+) without high-risk factors, treatment options may include chemotherapy combined with any of the following: selective CRT (determined by tumor response to chemotherapy), neoadjuvant long- or short-course CRT, or TNT [9,90,95,96].

For T2N1 and T3N0,1 patients, administering neoadjuvant chemotherapy using FOLFOX, followed by radiation with sensitizing fluoropyrimidine (5FUCRT) only when the primary tumor reduction is less than 20%, or chemotherapy is stopped because of adverse effects, may reduce the risk of radiotherapy-related complications [97]. In the PROSPECT trial, FOLFOX with 5FUCRT, indicated only when the tumor decreased in size by less than 20%, was non-inferior to 5FUCRU in terms of 5 year DFS (80.8% versus 78.6%, respectively) [98]. In the FOLFOX arm, 9.1% of patients received 5FUCRT preoperatively and 1.4% postoperatively. However, only patients eligible for sphincter-saving surgery were included in the study, and excluded patients included those with T4 tumors, ≥4 lymph nodes with short axis > 10 mm, or a tumor closer than 3 mm from the circumferential margin [98].

The PRODIGE 23 study indicated that a treatment sequence of neoadjuvant mFOLFIRINOX, chemoradiotherapy (CRT), surgery, and adjuvant chemotherapy resulted in significant improvements in patients with locally advanced rectal cancer compared with the conventional approach of preoperative CRT, surgery, and adjuvant chemotherapy [99]. This new approach demonstrated better outcomes across multiple measures: 5 year DFS, OS, metastasis-free survival, and cancer-specific survival, with favorable differences of 7.6%, 6.9%, 9.9%, and 5.7%, respectively. These findings indicate that this intensified treatment approach could potentially change clinical practice by offering better outcomes with manageable toxicity [99,100,101,102]. The ypCR rate was significantly higher in Arm B (27.5%) than in Arm A (11.7%) (*p* < 0.001) [99].

The RAPIDO study examined the effectiveness of a short course of radiation therapy followed by chemotherapy as a neoadjuvant treatment for locally advanced rectal cancer (cT4a/b, EMVI, cN2, mesorectal fascia involvement, or enlarged lateral lymph nodes) [103]. The RAPIDO trial supports short-course radiotherapy followed by chemotherapy over standard chemoradiation before TME for high-risk locally advanced rectal cancer, considering the effectiveness of this regimen in reducing distant metastases (19.8% vs. 26.6%, *p* = 0.004) and improving pathological response rates (27.7% vs. 13.8%, *p* < 0.001). However, a higher risk of locoregional recurrence (8.7% compared with 6.0%, *p* = 0.10) and the absence of a benefit in overall survival indicated that individualized approaches are necessary to balance systemic and local control [103]. This study revealed a significant decrease in 3 year disease-related treatment failure in the experimental group (23.7%) compared with the standard group (30.4%), suggesting better systemic control [104,105].

For cancers of the upper rectum (11–15 cm from the anal margin), surgical intervention should generally be the primary treatment approach, as neoadjuvant chemoradiotherapy typically offers no significant advantages [8,92]. Preoperatively, the possibility or certainty of a temporary or permanent stoma, its implications, and its management should be discussed with patients. Different stoma sites should be marketed preoperatively after discussions between surgeons, nurses/stoma therapists, patients, and/or family members [91].

After optimal MRI staging, patients with resectable cancer not threatening the mesorectal fascia (T2-T4peritoneum, N0-2, M0, and mesorectal fascia not threatened) may be approached by surgical resection. When high-risk factors for local recurrence are revealed by MRI (T3c disease—tumor extending > 15 mm from the muscularis propria, tumor deposits, positive mesorectal lymph nodes, and EMVI+), neoadjuvant therapy should be used [10,106].

### 3.8. Evaluation of Tumor Response After Neoadjuvant Therapy

Following the completion of neoadjuvant treatment, the tumor response should be reassessed [92].

In recent years, the TNT strategy has improved the complete response rate in patients with locally advanced rectal cancer. The pCT in surgical patients or sustained cCR for at least 12 months in those undergoing NOM increased to 36–38%, which was significantly higher than the 21% observed with conventional preoperative chemoradiotherapy [107,108].

Tumor response to neoadjuvant therapy significantly impacts long-term prognosis, with a 5 year DFS of 83.3% in patients with pCR compared with 65.6% in those without pCR [109]. Therefore, evaluating the efficacy of preoperative treatment (with MRI and endoscopy playing key roles) is essential for informing subsequent treatment strategies and estimating patient outcomes.

Magnetic resonance imaging (MRI) is the preferred modality for evaluating rectal cancer response because of its superior soft tissue contrast and capacity to assess both morphological and functional alterations. It facilitates tumor staging, evaluation of fibrosis and mucinous changes, and assessment of circumferential resection margins and extramural vascular invasion, which are critical factors for prognosis and recurrence risk [110,111,112]. Diffusion-weighted and perfusion MRI can predict tumor aggressiveness and residual tumor extent [110,113].

Endoscopic evaluation, often combined with MRI, provides a reliable estimation of tumor response. However, both methods have limitations and should be included in a multidisciplinary approach [114].

Neoadjuvant therapy is widely recommended for middle and lower rectal cancers, with a wide variety of tumor responses ranging from cCR and pCR to minimal response or even tumor progression [115]. Approximately 20% of patients with rectal cancer exhibit poor responses to neoadjuvant therapy. In this subset of patients, surgical delay is associated with adverse oncological outcomes; therefore, clinical and radiological evaluations of the treatment response are recommended at four weeks [115].

The first assessment was performed to determine complete clinical response (cCR) and eligibility for NOM 1–3 months after TNT completion [9]. cCR refers to the inability to detect any remaining tumor through clinical examination after neoadjuvant treatment [116,117,118]. To evaluate cCR, a combination of methods, including digital rectal examination, rectoscopy, and MRI, should be used [104,105,106]. The criteria used to define cCR were: (a) no palpable tumor on digital rectal examination; (b) absence of visible tumor on rectoscopy, with no residual superficial ulceration, no irregularity, no nodules, with only whitening of the mucosa and teleangiectasia with mucosa integrity being acceptable [116,118]; (c) MRI—significant downsizing with no remaining tumor; only residual fibrosis on diffusion-weighted imaging, and no suspicious lymph nodes [9]. In the OPRA trial, MRI variables associated with unfavorable tumor biology in the multivariate analysis were tumor involvement of the mesorectal fascia, presence of EMVI, and involvement of mesorectal lymph nodes [119].

Endoscopic biopsy is not mandatory and is associated with a high false-negative rate for assessing the presence of residual cancer after neoadjuvant therapy [92].

The timing to assess tumor response is not earlier than 8 weeks after radiotherapy in patients receiving induction chemotherapy and within 4 weeks after chemotherapy in patients receiving consolidation chemotherapy [61,120,121,122].

### 3.9. Non-Operative Management

Non-operative management may be discussed during the informed consent process for selected patients who demonstrate cCR after neoadjuvant therapy. This approach is appropriate in centers with experienced multidisciplinary teams and established protocols and for patients who are capable and compliant with intensive surveillance [9,61,92,123].

Patients who achieve cCR and consider NOM as an option instead of proctectomy should be informed about the risk of recurrence, which is approximately one-third of patients. Notably, there are currently no reliable prognostic indicators that can predict NOM failure [62]. The outcomes of salvage operations for tumor recurrence or regrowth following NOM should be included in the informed consent process.

The Organ Preservation in Patients with Rectal Adenocarcinoma Treated with Neoadjuvant Therapy (OPRA) trial included 324 participants diagnosed with stage II and III rectal cancer. The randomization was for induction chemotherapy followed by chemoradiation (CHT-RT), whereas the other group underwent chemoradiation followed by consolidation chemotherapy (RT-CHT) [124]. The authors concluded that organ preservation was achieved in approximately 50% of cases after total neoadjuvant therapy [94]. This approach demonstrated no apparent negative impact on survival compared with historical groups treated with a combination of chemoradiotherapy, TME, and subsequent chemotherapy [94].

Following TNT, patients were evaluated for treatment effectiveness using digital rectal and endoscopic examinations after 8–12 weeks [125]. Clinical response assessment, available for 294 patients, revealed the following: cCR, 124; nCR, 113; iCR, 57 patients [126]. Patients with complete or near-complete response to TNT had the option of NOM, whereas those with incomplete response were recommended rectal resection [125]. The TME rate was 21% in patients with cCR and 48% in those with nCR [126].

With a median follow-up period of 5.1 years, the 5-year DFS rates were 71% and 69% (*p* = 0.68) for CHT-RT and RT-CHT, respectively. TME-free survival rates were 39% and 54% (*p* = 0.012) for the CHT-RT and RT-CHT groups, respectively. Tumor regrowth was observed in 81 patients (94% within 2 years and 99% within 3 years). There was no difference in DFS between TME after restaging and after regrowth (64% versus 64%, *p* = 0.94) [124].

Patients opting for NOM should be surveyed in addition to the standard protocol for detecting tumor recurrence using an additional program to detect tumor regrowth.

Patients opting for NOM should undergo an additional surveillance program, including (a) digital rectal examination and flexible rectosigmoidoscopy at three-month intervals during the first two years and every six months for the following three years, and (b) pelvic MRI every six months for three years, and annually for the following two years [9,61].

### 3.10. Immunotherapy

For patients with MSI-H or dMMR tumors, immunotherapy using checkpoint inhibitors is recommended for up to six months [9,61].

Checkpoint inhibitors interfere with the mechanisms that downregulate immune responses, thereby amplifying the ability of the immune system to target and eliminate cancer cells. This is particularly effective in MSI-H/dMMR tumors because of their high mutational burden and the presence of tumor-infiltrating lymphocytes [127,128,129].

In 2022, researchers from the Memorial Sloan Kettering Cancer Center, New York, presented a study involving 12 patients with locally advanced, mismatch repair-deficient rectal cancer treated with single-agent dostarlimab and an anti-programmed death 1 (PD-1) monoclonal antibody [130]. At 12 months following clinical examination, MRI, PET-CT, endoscopy, and biopsy, all patients maintained cCR [130].

Immunotherapy has demonstrated significant efficacy in patients with MSI-H or dMMR rectal cancer. These patients often respond well to immune checkpoint inhibitors, which can lead to high rates of tumor regression and organ preservation [131,132]. However, patients with microsatellite stability (MSS) generally have poor responses to immunotherapy alone, necessitating combination strategies with other treatments [131,133].

The effectiveness of ICIs in MSI-H/dMMR rectal cancer is attributed to the unique molecular features of these tumors, including mutations that generate neoantigens. These neoantigens facilitate T cell activation and enhance antitumor immunity, making ICIs particularly effective [134].

ICIs modify the tumor microenvironment by promoting immune cell infiltration, particularly CD8+ T cells, and stimulating the release of anticancer cytokines [135]. These ICIs target specific immune checkpoints, including PD-1 and CTLA-4, resulting in an amplified immune response against cancer cells.

Recent studies have explored the integration of immunotherapy with traditional treatments, such as chemotherapy and radiotherapy, to enhance therapeutic outcomes.

ICIs have shown significant efficacy in patients with dMMR rectal cancer, with high rates of cCR and pCR in patients treated in the neoadjuvant setting (74% achieved cCR, 80% of those with resection had pCR, and no disease progression during follow-up) [136,137].

Grewal et al. reviewed the results of 16 patients treated with a single agent, programmed death 1 (PD-1) blocker, and three patients treated with a dual blockade (PD-1 blocker and anti-cytotoxic T-lymphocyte antigen 4, CTLA-4). cCR was reported in 14 of 19 patients (74%), and pCR was reported in 80% of cases (4 of 5 patients who underwent surgery). All patients were progression-free at the 6–12 month follow-up [136].

Combining immunotherapy with neoadjuvant chemoradiotherapy has shown promise, particularly in MSS rectal cancer, by potentially enhancing the complete clinical response rate and improving the quality of life [131,133]. The INNATE trial investigated the addition of anti-CD40 agonists to standard neoadjuvant therapy, demonstrating their feasibility and potential for improved pathologic complete response rates [138,139].

Li et al. analyzed a cohort of 25 patients with high-risk pMMR rectal cancers receiving TNT (induction chemoimmunotherapy, three cycles of oxaliplatin and capecitabine + camrelizumab, followed by long-course CRT, 50.6 Gy radiotherapy + capecitabine, followed by consolidation chemotherapy, and two cycles of oxaliplatin and capecitabine) [140]. The pCR rate was 33.3% (7/21), cCR was 48%, a major pathological response was observed in 38.1% (8/21) of the cases, and R0 was achieved in 21/21 cases [140].

Similar results were observed by Yang et al. in 50 patients with locally advanced pMMR/MSS rectal tumors when evaluating the efficacy and safety of the PD-1 blockade plus long-course CRT [141]. The pCR was 40%, with tumor regression grades of 1, 2, and 3 in 30%, 18%, and 4% of patients, respectively [141].

Radiotherapy may increase the expression of immune targets, such as PD-L1, potentially increasing the efficacy of immunotherapy and resulting in synergistic effects [142,143]. This approach is particularly promising for older patients or those who are unfit for surgery, offering a non-surgical treatment pathway [143].

Neoadjuvant immunotherapy does not adversely affect surgical outcomes, suggesting that it can be safely integrated into treatment protocols without increasing the risk of postoperative complications [144]. An analysis of the National Cancer Database between 2010 and 2020 revealed that 126 of 26,229 patients received immunotherapy in addition to CRT as neoadjuvant therapy [144]. They were propensity-matched into two groups of 125 patients each (immunotherapy + CRT + surgery versus CRT + surgery). Surgical resection was performed later in the immunotherapy cohort (245 vs. 144 days, *p* < 0.001), with no statistically significant differences in hospital stay (5 vs. 5 days), 30 day readmissions (7 vs. 9, *p* = 0.617), and 30 day mortality (0 vs. 1, *p* = 1) [144].

Treatment with ICIs can lead to pseudoprogression and pseudoresidue, where initial radiological evaluations may suggest disease progression, but subsequent assessments confirm a complete pathological response. This phenomenon was observed in 23.1% and 76.9% of patients, respectively, highlighting the need for careful evaluation [137].

The presence of pseudoprogression (described in 23.1% of cases) and pseudoresidue (described in 76.9% of cases) are particular aspects in rectal cancer patients with MSI-high/dMMR after neoadjuvant immune checkpoint inhibitors [137]. Thus, the development of specific strategies for response evaluation is required to accurately identify patients who may benefit from organ-preserving approaches [137].

Considering that MSI testing is limited by intratumoral heterogeneity, advanced imaging techniques, such as multiparametric MRI, are being developed to assess the MSI status preoperatively, which could aid in treatment planning and in guiding biopsy [59].

## 4. Discussions

In this review, we thoroughly examined the current evidence on preoperative care to inform the management of patients with rectal cancer.

High-volume centers and multidisciplinary teams (MDTs) are crucial for the optimal multimodal management of these patients. Extensive evidence links hospital volume to improved surgical quality and oncologic outcomes. A recent multicenter study of nearly 17,000 colorectal cases found that low-volume hospitals had higher rates of severe postoperative complications and suboptimal oncologic surgery than very high-volume centers [145]. The centralization of rectal cancer care was associated with more frequent use of indicated neoadjuvant therapy and adequate lymph node yields, reflecting better adherence to quality standards [145]. A post hoc analysis of a rectal cancer trial showed that hospitals with high MDT quality (≥5 specialist disciplines, weekly meetings, >200 cases/year) achieved significantly higher 3 year OS (90.5% vs. 78.1%) in locally advanced disease [146]. MDT discussions ensure proper staging, consistent application of best practices, and individualized treatment plans, thereby increasing curative-intent resection rates and reducing unwarranted variability in care [147]. Reflecting this, contemporary guidelines strongly recommend that rectal cancer patients be managed with input from an experienced MDT tumor board [95]. Concentrating care in high-volume centers with skilled MDTs promotes adherence to evidence-based pathways and improves survival and functional results.

Despite therapeutic advances, many patients face barriers in accessing the full rectal cancer care pathway, from timely diagnosis to multimodal treatment and surveillance, leading to outcome disparities. Socioeconomic and geographic factors often impede prompt biopsy, neoadjuvant chemoradiotherapy, surgery, and appropriate follow-up. For example, in the United States, disadvantaged groups have higher colorectal cancer mortality rates, reflecting care inequities [148]. A national database analysis showed that Hispanic and Black patients with rectal cancer experience significant delays in time to surgery, radiation, and chemotherapy compared with others, with a median time to surgery of 94.2 days for Hispanic versus 79.1 days for non-Hispanic patients [149]. Globally, low-resource regions may lack high-quality MRI staging or radiotherapy facilities, resulting in more advanced disease at presentation and lower survival rates. These challenges highlight the need for improved infrastructure and referral networks to ensure that all patients receive guideline-concordant therapy (neoadjuvant treatment, expert surgery, and timely follow-up) without delays. Bridging these gaps is critical to reduce global inequities in rectal cancer outcomes.

The primary clinical implication of the current study is the emphasis on the necessity of precise staging and risk stratification using high-resolution pelvic MRI and advanced imaging. These methods are crucial for accurate staging and guiding treatment decisions. This study highlights TNT as the new standard of care for locally advanced rectal cancer, demonstrating improved tumor response rates and the potential for organ preservation. In alignment with emerging evidence, this study underscores the importance of assessing MSI status to identify patients who may benefit from immunotherapy, as well as the role of standardized genetic testing, which holds significant prognostic and predictive value. Furthermore, the role of multidisciplinary tumor boards in optimizing rectal cancer management and enhancing patient outcomes is detailed.

This review has several limitations. First, the included studies exhibited heterogeneity in terms of patient populations, treatment protocols, and endpoints. Variability in the definition of multimodal therapy and differences in follow-up duration complicate direct comparisons between studies. Many published analyses focus on oncologic endpoints but underreport functional outcomes and patient-reported QoL, limiting the ability to draw firm conclusions regarding QoL across strategies. Notably, a recent systematic review found that none of the available studies on advanced and recurrent rectal cancer reported functional outcomes, and only a few reported QoL outcomes using inconsistent metrics [150]. Second, overlapping data across reviews and potential publication biases must be acknowledged. Well-performing centers are often over-represented, whereas results from low-resource settings are limited. Several pivotal trials have short-term results available but limited 5-year follow-up, and real-world data from developing regions are lacking. These factors reduce the generalizability of our conclusions. Ongoing studies and future standardized reporting with a core outcome set for oncologic and QoL measures are needed to address these gaps.

## 5. Conclusions

Multimodal preoperative management of rectal cancer requires a comprehensive, multidisciplinary strategy to achieve optimal therapeutic outcomes. Precise tumor staging utilizing sophisticated imaging techniques, particularly high-resolution pelvic MRI and endoscopic ultrasound, is fundamental for accurate risk stratification and treatment planning. Total neoadjuvant therapy, which combines chemoradiotherapy with systemic chemotherapy, has established itself as the standard of care for locally advanced rectal cancer (T3, T4, and N+), demonstrating improved tumor response rates and enabling the potential for organ-sparing treatment. Additionally, the integration of personalized treatment regimens, informed by molecular profiles such as microsatellite instability and mismatch repair status, provides significant opportunities for individualized care, notably through the application of immune checkpoint inhibitors. Ongoing progress in molecular diagnostics, targeted therapeutic approaches, and imaging advancements highlight the necessity of a personalized, multidisciplinary approach to enhance patient prognosis and quality of life.

## Figures and Tables

**Table 1:** Comparative analysis of the international rectal cancer management guidelines.

	ESMO [11,12]	NCCN [13]	ASCO [9]	JGCRS [14]	CSCO [15]
Staging, after diagnosis	MRI: high-risk criteria—cT4a, cT4b, mesorectal fascia involvement, ≥4 LNs, EMVI+, lateral LNs ≥ 7 mm. ERUS (cT1 vs. cT2) CT chest and abdomen	Pelvis MRI ERUS—only if MRI contraindicated Chest and abdomen CT (MRI if allergy or kidney failure)	High-resolution pelvic MRI with a standardized report. Risk factors: T4, EMVI, tumor deposits, threatened MRF/intersphincteric plane.		High-resolution pelvic MRI Transrectal ultrasound CT chest and abdomen
MDT	Oncologists, radiologists, surgeons, radiation oncologists, pathologists—guidelines for decision-making.	Experienced MDT	Medical oncologists, surgical oncologists, radiation oncologists, radiologists, pathologists, gastroenterologists—guideline for treating patients.		Composition: colorectal surgery, hepatobiliary surgery, medical oncology, radiation oncology, imaging. Cases to be discussed: patients with mid and low rectal tumors; patients with liver metastases only; patients with potentially resectable metastases, Fixed disciplines, time each week, location, equipment.
Restaging after neoadjuvant therapy	MRI, endoscopy, digital rectal examination. ERUS not recommended. Biopsies not recommended in surveillance for cCR.	Chest and abdomen CT Pelvis MRI	In the setting of NOM: DRE, flexible sigmoidoscopy, rectal MRI.		High-resolution MRI
Genetic testing recommendations	Localized tumors: - MSI and/or MMR in all patients at diagnosis - No recommendations for RAS, BRAF V600E, NTRK, HER2. In metastatic stages: - MMR status and BRAF mutations at all pts. - RAS and BRAF V600E testing mandatory before anti-EGFR therapy - HER2 amplifications in RAS-wt - NGS availability: KRAS, G12C, POLE, NTRK fusions, RET fusions, TMB-H	Without suspected metastasis: - MMR/MSI testing - PIK3CCA With suspected metastasis: - RAS, BRAF, HER2, MMR/MSI - POLE/POLD1, RET, NTRK	Locally advanced cancers—MSI or MMR prior to treatment starting.	RAS (KRAS, NRAS) and BRAF V600E MSI/MMR status	On the radical surgery specimen: MMR proteins/MSI. Grade II: RAS and BRAF gene mutations. Metastatic CRCs: MMR protein/MSI, RAS, BRAF gene mutations.
Neoadjuvant therapies	CRT: lower and middle rectal tumors, cT2N+, cT3No, cT3N1. When organ preservation is intended, dose escalation with endorectal brachytherapy. TNT: upper third cT4 or involved MRF tumors; middle and lower rectum for high-risk tumors. CHT: inclusion according to the PROSPECT trial criteria. Not recommended for dMMR and MSI-H tumors. Cannot be recommended NOM in cases with cCR. Immunotherapy	pMMR/MSS, T3Nany; T1,2N1,2; T4Nany: - Long course CRT/RT, followed by CHT - Short-course RT followed by CHT - CHT followed by long- or short-course CRT - CHT, followed by long- or short-course CRT in cases with tumor regression less than 20%	TNT indicated: - in locally advanced low rectal tumors - patients at higher risk of local or distant metastases—presenting high-risk factors CHT according to the PROSPECT study conditions	Neoadjuvant RT: cT3 or deeper, or N+ Preoperative CRT is recommended in cases with a high risk of local recurrence. Efficacy of preoperative CHT has not been established. CRT is indicated for unresectable locally advanced and recurrent tumors. Neoadjuvant chemotherapy indication for resectable liver metastasis was not established.	cT1,2N+, cT3,4Nany—concurrent CRT± interval CHT (reassessment)

ASCO: American Society of Clinical Oncology; CT: computed tomography; CSCO: Chinese Society of Clinical Oncology; EMVI: extramural vascular invasion; CRC: colorectal cancer; CRT: chemoradiotherapy; CHT: chemotherapy; DRE: digital rectal examination; ESMO: European Society of Medical Oncology; ERUS: endorectal ultrasound; JGCRS: Japanese Society for Cancer of the Colon and Rectum; LN: lymph node; MDT: multidisciplinary team; MSI: microsatellite instability; MMR: mismatch repair; NGS: next-generation sequencing; MRF: mesorectal fascia; MRI: magnetic resonance imaging; NCCN: National Comprehensive Cancer Network.

## Data Availability

All data generated or analyzed during this study are included in this article. Further inquiries can be directed to the corresponding author.
